# Variable Expression of Oncogene-Induced Senescence/SASP Surrogates in HPV-Associated Precancerous Cervical Tissue

**DOI:** 10.3390/cimb46120818

**Published:** 2024-12-02

**Authors:** Tareq Saleh, Nisreen Himsawi, Amani Al Rousan, Ahmad Alhesa, Mohammed El-Sadoni, Suzan Khawaldeh, Nisreen Abu Shahin, Ala’ Abu Ghalioun, Bayan Shawish, Kholoud Friehat, Moureq R. Alotaibi, Ola Abu Al Karsaneh, Anas Abu-Humaidan, Rame Khasawneh, Ashraf I. Khasawneh, Sofian Al Shboul

**Affiliations:** 1 Department of Pharmacology and Public Health, Faculty of Medicine, The Hashemite University, Zarqa 13133, Jordan; 2 Department of Microbiology, Pathology and Forensic Medicine, Faculty of Medicine, The Hashemite University, Zarqa 13133, Jordan; 3 King Hussein Medical Center, Royal Medical Services, Amman 11942, Jordan; 4 Department of Pathology, Microbiology and Forensic Medicine, School of Medicine, The University of Jordan, Amman 11942, Jordan; 5 Department of Pharmacology and Toxicology, College of Pharmacy, King Saud University, Riyadh 12271, Saudi Arabia; 6 Department of Laboratory Medicine, National Institutes of Health Clinical Center, Bethesda, MD 20892, USA

**Keywords:** oncogene-induced senescence, SASP, cervix, premalignant, HPV

## Abstract

Oncogene-induced senescence (OIS) is a form of cellular senescence triggered by oncogenic signaling and, potentially, by infection with oncogenic viruses. The role of senescence, along with its associated secretory phenotype, in the development of cervical cancer remains unclear. Additionally, the expression of the senescence-associated secretory phenotype (SASP) has not yet been explored in cervical premalignant lesions infected by the Human Papilloma Virus (HPV). This study aimed to investigate the expression of OIS and SASP markers in HPV-infected cervical precancerous lesions. We used a set of patient-derived precancerous (*n* = 32) and noncancerous (chronic cervicitis; *n* = 10) tissue samples to investigate the gene expression of several OIS (*LMNB1*, *CDKN2A*, *CDKN2B*, and *CDKN1A*), and SASP (*IL1A*, *CCL2*, *TGFB1*, *CXCL8*, and *MMP9*) biomarkers using qRT-PCR. OIS status was confirmed in precancerous lesions based on Lamin B1 downregulation by immunohistochemical staining. HPV status for all precancerous lesions was tested. Most of the noncancerous samples showed high Lamin B1 expression, however, precancerous lesions exhibited significant Lamin B1 downregulation (*p* < 0.001). Fifty-five percent of the precancerous samples were positive for HPV infection, with HPV-16 as the dominant genotype. Lamin B1 downregulation coincided with HPV E6 positive expression. *CDKN2A* and *CDKN2B* expression was higher in precancerous lesions compared to noncancerous tissue, while LMNB1 was downregulated. The SASP profile of premalignant lesions included elevated *CXCL8* and *TGFB1* and reduced *IL1A*, *CCL2*, and *MMP9*. this work shall provide an opportunity to further examine the role of OIS and the SASP in the process of malignant cervical transformation.

## 1. Introduction

Cervical cancer is the fourth most prevalent type of cancer among women in the world [1]. Additionally, the survival rate for cervical cancer patients in the United States (US) during the period from 2013 to 2019 was 67%, so there is scope for improved therapeutic approaches [2]. Human papillomavirus (HPV) infection is the most common cause of cervical cancer [3]. Precancerous lesions of the cervix are abnormal changes in cervical epithelial cells that have the potential to develop into cervical cancer [4]. Cervical intraepithelial neoplasia (CIN) is a precancerous lesion that is classified as low-grade (CIN1) and high-grade (CIN2 or CIN3) [5]. Unfortunately, the majority of high-grade CIN can progress into full-blown cervical cancer if left unmanaged [6]. CIN is closely associated with persistent infection by high-risk HPV, with more than 90% of high-grade CIN cases being positive for HPV [7].

The HPV genome consists of three functional regions, including the early (E) region. Several oncoproteins are present in this region, including E2, E6, and E7 [8]. E6 and E7 interfere with apoptosis induction in host cells, assisting in providing a suitable environment for virus replication [8]. Following the infection of a cervical epithelial cell, the HPV genome fuses with the host DNA in high-grade CIN, disrupting the E2 gene; thus, E6 and E7 expression is increased [9]. Degradation of the tumor suppressor protein p53 by E6, and retinoblastoma (pRb) by E7, leads to the deregulation of the growth cycle in the host cell [10]. As a result of the degradation of p53 by E6 via its binding to the E6-associated protein (E6AP), inhibitory control over the cell cycle is lost [11]. Degradation of pRb by E7 liberates the E2F transcription factor and allows the host cell to enter the S phase, further facilitating the viral replication cycle [11]. Inactive p53 and pRb proteins can lead to genomic instability and an accelerated rate of proliferation. Transformation is more likely to occur as a result of the higher rate of replication due to the increasing amount of damaged DNA that cannot be repaired [12]. The major impact of HPV on cell cycle regulation and its connection to cervical cancerous transformation has suggested the role of cellular senescence in the development of cervical cancer [13].

Senescence is an established hallmark of cancer [14]. Oncogene-induced senescence (OIS) is a form of senescence that characterizes precancerous lesions [15,16]. OIS represents a durable form of cell cycle arrest that hampers the proliferation of cells with oncogenic potential [17]. Previous evidence has suggested a link between senescence and HPV infection as E6 inhibition, coupled with increased p53 expression and upregulation of its downstream cyclin-dependent kinase inhibitor, p21^Cip1^, lead to senescence induction [13]. Moreover, in HPV-positive tumor cells, suppression of E7 expression leads to the rapid induction of senescence and permanent proliferative arrest in vitro [13,18]. Unfortunately, an examination of OIS in HPV-infected precancerous cervical tissue has not been performed in vivo.

Moreover, the senescence-associated secretory phenotype (SASP), the functional arm of cellular senescence, is involved in propagating the progression of precancerous lesions, although in a complex manner [19]. The SASP consists of a variety of pro-inflammatory cytokines, chemokines, growth factors, and proteases secreted by senescent cells, many of which can have pro-tumorigenic potential [20]. While SASP can reinforce tumor suppression by promoting immune clearance of precancerous cells [21], it can also favor a pro-tumorigenic microenvironment that is largely dependent on its secreted factors’ spectrum [22]. For example, SASP factors, such as IL-6, IL-8, and VEGF, promote cell proliferation, angiogenesis, and inflammation, creating conditions permissive for malignant transformation [23]. More importantly, the SASP is transcriptionally and functionally heterogeneous [24,25]. The variability in SASP’s composition is trigger- and tissue-dependent and can result in differing effects on surrounding cells, leading to either tumor suppression or to tumor promotion.

This study aimed to analyze the expression profile of four OIS-associated markers (*LMNB1*, *CDKN1A*, *CDKN2A*, and *CDKN2B*) and five SASP factors (*MMP9*, *IL1A*, *CCL2*, *CXCL8*, and *TGFB1)* in precancerous samples of the cervix. The study included both precancerous and noncancerous samples, which were tested for HPVgenotypes. These samples were specifically associated with high-risk HPV infection.

## 2. Materials and Methods

### 2.1. Tissue Samples

The sample comprised formalin-fixed paraffin embedded (FFPE) tissue blocks of 32 precancerous cervical lesions and 10 cervical samples with active chronic cervicitis (used as noncancerous controls), which were obtained from Jordanian Royal Medical Services (JRMS), Amman, Jordan. The inclusion criteria for sample collection included: all cervical precancerous grades, regardless of their HPV status, and women over the age of 18 years. The exclusion criteria include patients whose age was less than 18 years. All research activities under this work were conducted based on ethical approval obtained by Institutional Review Boards (IRB) at the Hashemite University (Protocol No. 11/9/2021/2022) and JRMS (Protocol No. 10/2022/10) in agreement with the standards of the Declaration of Helsinki. The requirement for informed consent was waived by the IRB at the primary institution of the principal investigator that approved the study (the Hashemite University) due to its nature, which strictly includes the use of surplus tumor tissue samples. This waiver was further confirmed by the IRBs of the clinical center where samples were collected (JRMS).

### 2.2. Sample Processing and RNA and DNA Extraction

Several 20 µm thick section ribbons from each FFPE sample block were carefully cut and harvested into carefully pre-labeled Eppendorf tubes for deparaffinization steps and RNA extraction using RNeasy FFPE Kit (QIAGEN, Hilden, Germany), as previously described [26]. The eluted RNA was measured using the Qubit 3.0 fluorometer (Thermo Fisher Scientific, Waltham, MA, USA). Then, the samples were stored at −80 °C for subsequent use. Another ribbon was harvested for DNA extraction purposes using the QIAamp DNA tissue kit (Qiagen, Hilden, Germany), following the instructions provided. DNA was eluted in 200 μL AE buffer and stored at −20 °C for subsequent processing. RNA and DNA extracted from the samples have been stored, enabling potential future genomic or transcriptomic analyses to further explore molecular mechanisms associated with HPV infection and cervical lesion progression.

### 2.3. HPV Detection and Genotyping

HPV detection and genotype identification were performed using the Real-time PCR (RT-PCR) thermocycler (Bioer, Hangzhou, China), as previously described (1). The REALQUALITY RQ-Multi HPV detection kit, an in vitro diagnostic kit (AB ANALITICA, Padova, Italy), was utilized for this purpose. This kit can identify 28 HPV types, including 14 high-risk (16, 18, 31, 33, 35, 39, 45, 51, 52, 56, 58, 59, 66, and 68), 6 potential high-risk (−26, −53, −67, −70, −73, and −82), and 8 low-risk (6, 11, 40, 42, 43, 44, 55, and 83) genotypes. If a sample tested positive for HPV 16, HPV 18, or any other high-risk or potentially high-risk genotype (received a single signal), it was classified as a single-genotype infection. If two signals were detected, it was considered a two-genotype infection, and if three signals were detected, it was classified as a three-genotype infection. The amplification targets a region in the HPV genome encompassing E6 and E7, with the genotypes detected collectively except for HPV-16 and HPV-18, which are individually identified. The kit incorporates internal, negative, and positive controls. The thermocycling conditions involved an initial step of UNG activation at 50 °C for 2 min, followed by initial denaturation at 95 °C for 10 min, and then 45 cycles consisting of denaturation at 95 °C for 15 s, annealing at 60 °C for 60 s, and extension at 72 °C for 60 s. Data were interpreted according to the manufacturers’ guidelines, as detailed previously [27].

### 2.4. Reverse Transcriptase (cDNA) and Real-Time Quantitative PCR

The resulting RNA was revised to cDNA using the QuantiTect Reverse Transcription Kit (QIAGEN, Hilden, Germany), according to the manufacturers’ recommendations. DNA concentration was measured using the Qubit 3.0 fluorometer (Thermo Fisher Scientific, Waltham, MA, USA). Then, the samples were stored at −20 °C for subsequent use. RT-PCR was performed through the use of QuantiTect SYBR Green PCR Kit (QIAGEN, Hilden, Germany) as a source of Sybr green fluorescent dye, and was used in several amplification steps to target the DNA for each precancerous versus noncancerous cervical sample. OIS-associated genes and SASP primers were used against the GAPDH reference gene primers, as shown in Table 1, with each RT-PCR reaction undergoing the following thermal amplification steps: initial denaturation at 95 °C for 15 min, followed by 40 cycles at 94 °C for 15 s, 57.5 °C for 30 s, and 72 °C for 30 s, with a final extension step at 72 °C for 5 min. All PCR runs were performed on (BIOER, China) an instrument to measure the specific gene expression of both OIS-associated genes and SASP genes, whether upregulated or downregulated. Gene expression levels were determined through the ∆∆Ct (cycle threshold) formula [28]. Initially, we conducted a comparative threshold cycle analysis (∆*Ct*) for the precancerous sample against its own GAPDH *CT* result, as in: Δ*Ct* = *Ct*_gene of interest_ − *Ct*_GAPDH_. We then computed the average ∆*Ct* for the precancerous samples, which was termed “CIN samples Δ*Ct*”. Noncancerous group specimens were subjected to a similar ∆*Ct* calculation between the gene of interest and GAPDH gene to establish a baseline reference value. Normalization of the resulting Δ*Ct* values was performed through another subtraction equation, yielding the double delta *Ct* (∆∆*Ct*) = Δ*Ct*_CIN samples_ − Δ*Ct*_control samples_. Subsequently, we calculated the relative quantification (*RQ*) of gene expression using the equation *RQ* = 2^−∆∆*Ct*^. To facilitate comparison across all samples and genes, we applied a logarithmic transformation to the *RQ* values. A negative control was included in each run for quality control.

### 2.5. Immunohistochemistry (IHC) for Lamin B1

Immunohistochemical (IHC) was performed, as previously described [35,36,37]. Briefly, tissue sections were deparaffinized using xylene and rehydrated using graded ethanol jars (100%, 95%, and 70%). The heat-induced epitope retrieval for Lamin B1 antibody was performed with sodium citrate buffer solution (pH 6.0). This was followed by washing twice with phosphate-buffered saline (PBS), incubation with 3% hydrogen peroxide for 10 min and 5% bovine serum albumin (BSA) blocking; slides were incubated at room temperature for 2 h for anti-Lamin B1 (1:600, Novus biologicals, Cat. No. NBP2–59783, clone 4001, Centennial, CO, USA). Tissue sections were washed three times in PBS and treated with ultraVIEW Universal HRP Multimer (Ventana Medical Systems, Inc., Roche, Tucson, AZ, USA), then washed twice using PBS and incubated in a dark place with 3,3′-diaminobenzidine chromogen as substrate buffer for 10 min, then washed and counterstained with hematoxylin. Finally, dehydrated, cleared slides were mounted with DPX and visualized under a light microscope (Olympus Optical, Tokyo, Japan). The protein expression of senescence-associated biomarker, Lamin B1, was assessed in our cervical tissue samples cohort. Downregulation of Lamin B1 is suggestive of senescence [38,39], and we have previously validated its reliability in identifying senescence in vivo across several cancer models such as breast cancer [36,40,41,42]. The median percentage of positively stained tumor cells for Lamin B1 was assessed by two independent pathologists at The Department of Pathology at JRMS (co-author: AAR) and The Department of Pathology, Microbiology and Forensic Medicine/Jordan University Hospital (co-author: NAS) using light microscopy (Olympus Optical, Tokyo, Japan) under 20 × and 40 × objective lenses and as described previously [36,42]. For Lamin B1 grouping of high and low, we used the mean Lamin B1 expression of noncancerous samples as a cutoff point to define senescence status, where Lamin B1 expression below 88% was considered downregulation, indicating a senescence-positive profile. Subsequently, samples with expression >88% were considered senescence-negative.

### 2.6. Immunofluorescence (IF) for Lamin B1 and HPV16 + HPV 18

Immunofluorescence (IF) staining was conducted as previously described [35,36]. Briefly, multiple tissue slides were deparaffinized using xylene and rehydrated using graded ethanol (100%, 95%, and 70%). Antigen epitope retrieval for Lamin B1 antibody was performed with sodium citrate buffer solution (pH 6.0). Subsequently, slides were washed twice with PBS and incubated in 5% BSA to block non-specific binding. Then, slides were incubated with anti-Lamin B1 (1:600, EPR8985(B), Abcam, Cambridge, UK) and anti-HPV16 E6 + HPV18 E6 (1:100, [C1P5], Abcam, Cambridge, UK) antibodies. Following that, slides were incubated with fluorescent secondary antibodies 1:500 (Alexa Flour 488, A21121 and Alexa Flour 546, A11035, Invitrogen) for 1 h at room temperature. Subsequently, slides underwent a 15-min PBS wash. To visualize cell nuclei, DAPI staining (1:1000, Thermo Scientific, product number 62248) was applied for 3 min at ambient temperature, followed by an additional 3-min PBS rinse. Anti-fade fluorescence mounting medium (Abcam, catalog number: ab104135, Cambridge, UK) was applied and slides were stored in light-protected containers at 4 °C. Images were acquired using a Zeiss LSM780 confocal microscope system (Carl Zeiss AG, Jena, Germany). For quantitative analysis, ten images were obtained from five different samples stained with both anti-HPV16 E6 + HPV18 E6 and Lamin B1 using confocal microscopy (Carl Zeiss AG, Jena, Germany) and manual quantification for all cells in each image was conducted.

### 2.7. Statistical Analysis

Data were initially entered and calculated on Excel sheets, which were later transformed into SPSS (version 25) for further analysis. Shapiro–Wilk showed that our data do not appear to be normally distributed, therefore, the non-parametric Mann–Whitney U test was performed. All reported p values were two-tailed and *p* ≤ 0.05 was considered statistically significant.

## 3. Results

### 3.1. Examining the Prevalence of HPV Subtypes in Precanerous Cervical Lesions

**Figure 1:**
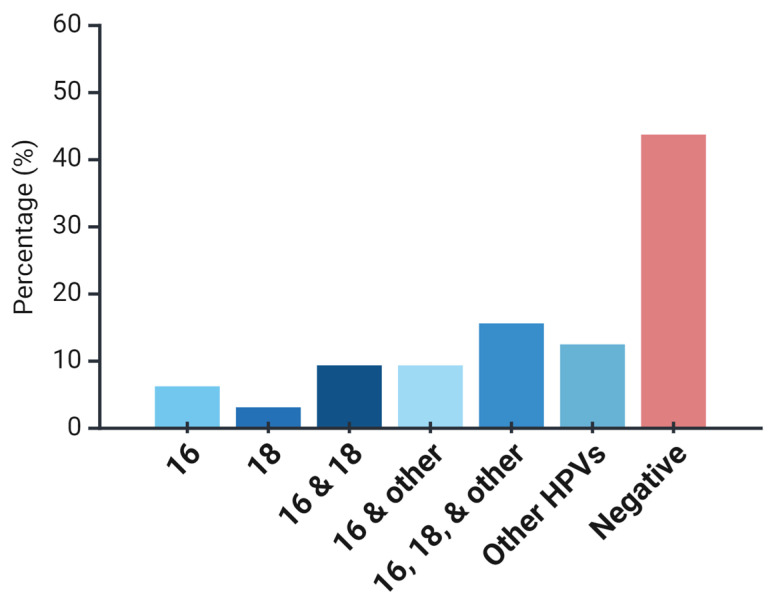
Distribution of HPV genotypes in cervical precancerous lesions. The bar chart shows HPV genotype distribution among cervical precancerous samples. Of these, 55% tested HPV-positive, with 78% associated with high-risk HPV subtypes. HPV-16 was the most common (72%), followed by HPV-18 (61%). Negative cases accounted for 45% of the sample. No variability metrics are shown as the data were derived from tabular representation.

Out of the total cases analyzed, 55% (*n* = 18/32) were found to be positive for HPV, with the majority of these cases (78%; *n* = 14/18) testing positive for high-risk (HR) HPV subtypes (Figure 1). Among the HR HPV-positive cases, HPV-16 was the most prevalent subtype, detected in 72% (*n* = 13/18) of the cases, followed by HPV-18, which was identified in 61% (*n* = 11/18) of the cases (Figure 1). The distribution of HPV infection patterns revealed that a single-genotype HPV infection was present in 17% of cases (*n* = 3/18), while multiple-genotype infections were observed in the remaining cases. Specifically, 33% (*n* = 6/18) exhibited two-genotype infections, 28% (*n* = 5/18) demonstrated infections involving three genotypes, and 22% (4/18) were infected with HPVs other than 16 or 18 (Figure 1). This indicates that a notable proportion of cases were infected with multiple HPV subtypes, highlighting the complexity of HPV involvement in CIN. Moreover, these findings underscore the dominance of HPV-16 and HPV-18 as HR HPV subtypes.

### 3.2. Examination of OIS Markers Expression in Cervical Precancerous Lesions

**Figure 2:**
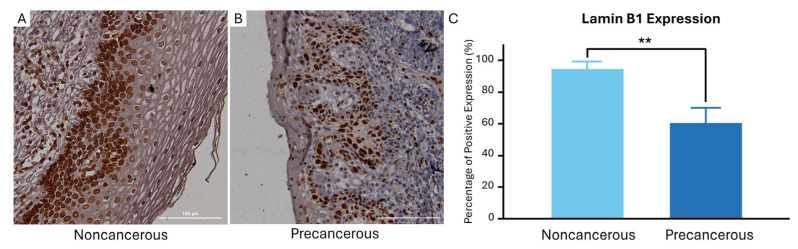
Immunohistochemical analysis of Lamin B1 expression in precancerous and noncancerous cervical tissue. A representative image of Lamin B1 expression in noncancerous cervical tissue (**A**) and (**B**) precancerous cervical tissues. (**C**) Quantification of Lamin B1-positive cells showing a significant reduction in precancerous cervical samples compared to noncancerous tissue (** *p* < 0.001). Data are presented as the median with 95% confidence intervals.

Our IHC analysis showed that Lamin B1 expression was significantly reduced in cervical precancerous samples (Figure 2A) relative to noncancerous tissue (Figure 2B). Specifically, the mean expression of Lamin B1 in CIN samples was mean 54% (range: 0–100% positive expression), in comparison to noncancerous tissue (*n* = 10), where the mean was 88% (range: 60–99% positive expression) (Figure 2C). Our comparison showed that most of the samples (75%; *n* = 24/32) exhibited reduced Lamin B1 expression in precancerous samples relative to noncancerous cervical tissue (*p* < 0.001), suggesting the development of OIS (Figure 2C).

**Figure 3:**
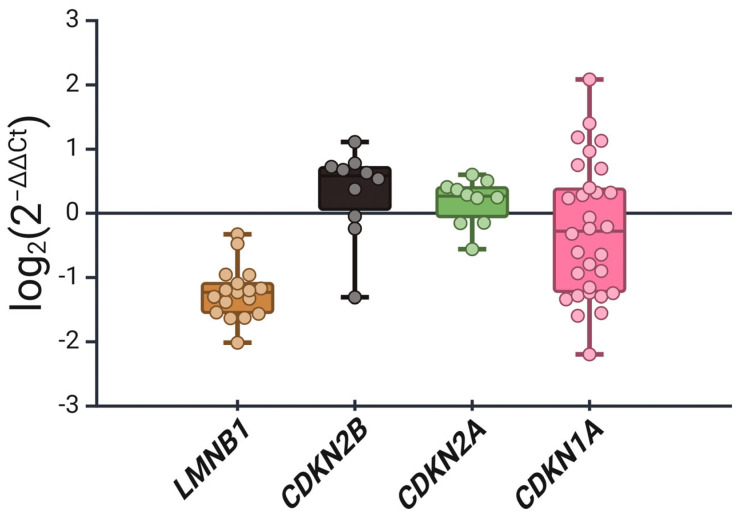
Fold change in mRNA expression levels of OIS markers in precancerous relative to noncancerous cervical tissue. *LMNB1* expression was significantly downregulated in precancerous lesions, confirming its immunohistochemically detected, reduced protein expression. In contrast, *CDKN2B* and *CDKN2A* (p15^INK4b^ and p16^INK4a^, respectively) were both significantly upregulated, indicating senescence activation. *CDKN1A* (p21^Cip1^) exhibited a variable expression pattern without consistent upregulation, suggesting its differential regulation in the precancerous state. Data are presented as log_2_ fold change, with each dot representing an individual (precancerous) sample. Created in BioRender.com.

We have further confirmed these findings by measuring the expression level of *LMNB1* in precancerous lesions relative to noncancerous lesions using RT-PCR (Figure 3). Similarly, *LMNB1* expression was significantly reduced in the precancerous lesions, which agrees with its downregulated protein expression level (Figure 3). Since the identification of senescence in vivo requires the measurement of several biomarkers, we measured the gene expression of three classical regulators of senescence, namely, p15^INK4b^ (*CDKN2B*), p16^INK4a^ (*CDKN2A*), and p21^Cip1^ (*CDKN1A*) [31,43]. p15^INK4b^ is an established cell cycle regulator and a valid senescence marker in precancerous tissue in vivo [16], and p16 ^INK4a^ is also a cell cycle regulator and is considered the gold-standard senescence protein marker [44]. Expectedly, the expression of both *CDKN2B* and *CDKN2A* was elevated in precancerous tissue when compared to noncancerous tissue, which confirms their positive senescence status (Figure 3). Interestingly, while we expected a similar profile for *CDKN1A*, another senescence-related cell cycle regulator [45], the expression pattern showed variability without a significant upregulation as per the other cell cycle regulators. Collectively, these data indicate that a senescence profile is more likely to be present in the precancerous state, which is consistent with the predominance of OIS in precancerous lesions [46,47].

**Figure 4:**
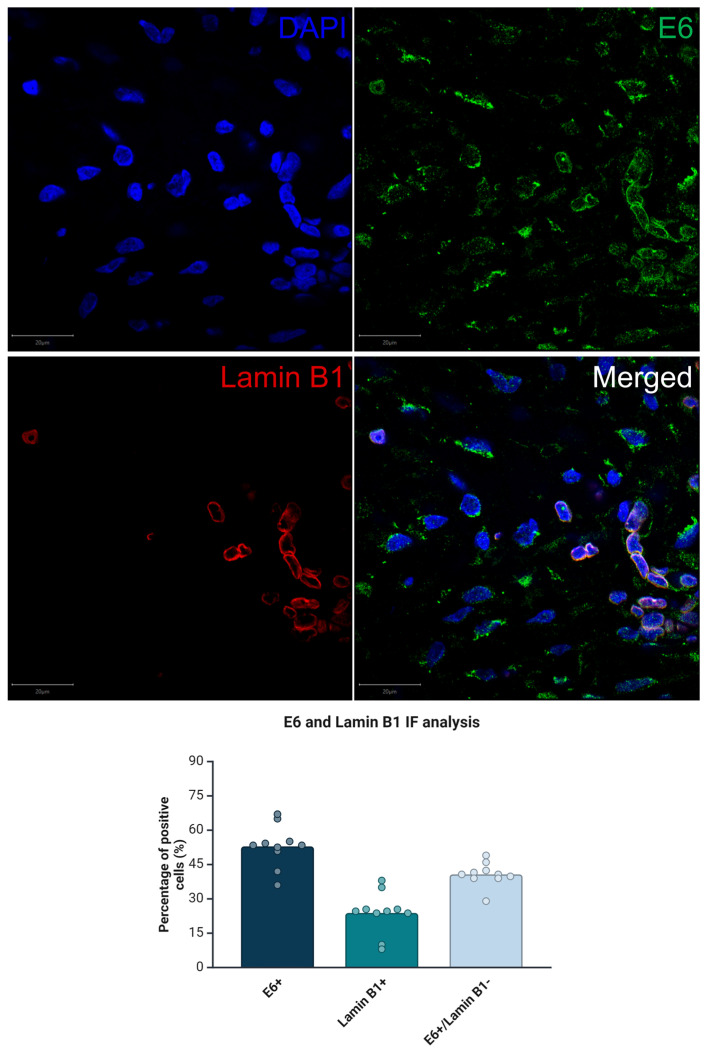
Co-immunofluorescent staining of HPV oncoprotein E6 and Lamin B1 in cervical precancerous lesions. Representative images show DAPI-stained nuclei (blue), Lamin B1 staining (red), E6 staining (green), and merged channels. The graph below quantifies the percentage of positive cells for E6, Lamin B1, and the co-localization of E6+/Lamin B1 cells. A total of 41% of the E6-positive cells exhibit downregulation of Lamin B1, suggesting that HPV E6 may be involved in driving the OIS phenotype in these lesions. Scale bar = 20 µm.

Lastly, and to confirm the connection between HPV infection and the OIS status of precancerous lesions, we measured the expression level of Lamin B1 in precancerous tissue samples as well as that of the HPV oncoprotein, E6, using co-immunofluorescent staining (Figure 4). Our data indicated that E6 was co-localized with downregulated Lamin B1 (E6-positive/Lamin B1-negative) in 41% of precancerous cells, suggesting that E6 might drive the OIS profile in premalignant cervical lesions.

### 3.3. Expression Variability of SASP Factors in Cervical Precancerous Lesions

**Figure 5:**
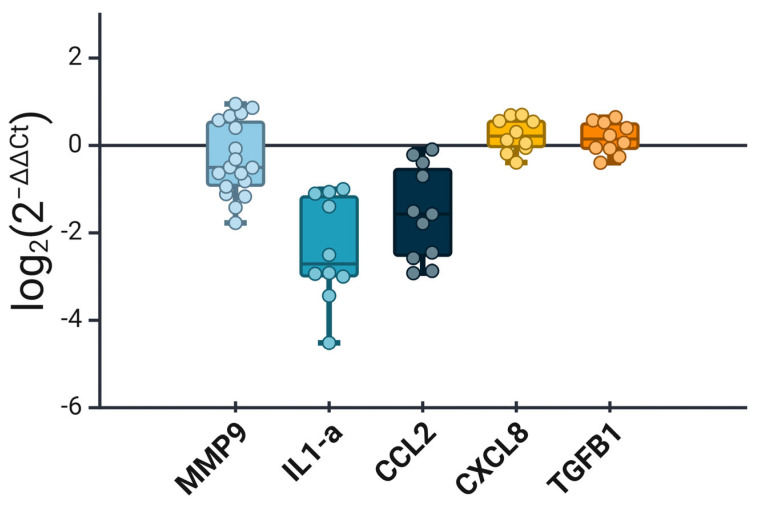
Gene expression analysis of SASP-related markers in cervical precancerous lesions compared to noncancerous inflammatory lesions. The box plot illustrates the fold change (log_2_) in expression of *MMP9*, *IL1A*, *CCL2*, *CXCL8*, and *TGFB1*. *IL1A* and *CCL2* expression was reduced in precancerous lesions, while *CXCL8* and *TGFB1* exhibited mild upregulation. *MMP9* expression was heterogeneous, with some samples showing upregulation and others showing downregulation. These data reflect the variability in SASP marker expression in precancerous lesions. Created in BioRender.com.

Next, we wanted to examine the expression level of an array of SASP-related genes in precancerous lesions and compare that with its corresponding level in noncancerous tissue. It is noteworthy that the noncancerous lesions were derived from cervical tissue with features of chronic inflammation. To our surprise, the expression of the SASP markers was variable and not as consistent as the other senescence-related markers (Figure 5). While *IL1A* and *CCL2* showed significantly reduced expression in the precancerous lesions, markers like *CXCL8* and *TGFB1* were elevated, albeit only slightly. *MMP9* showed observable heterogeneity, with many precancerous samples showing upregulated expression while the majority showed downregulated expression when compared to the inflammatory noncancerous lesions. These data indicate a high degree of variability in SASP markers’ expression in cervical precancerous lesions.

## 4. Discussion

Our analysis first provided insights into the rate of HPV infection in cervical precancerous lesions. Intrinsically, the distribution of multiple HR-HPV infections varies among different cervical diseases and their clinical significance remains controversial [48]. Recently, Zhong et al. examined multiple HR-HPV infections in different cervical lesions and found that multiple infections occurred more frequently in CIN compared to squamous cervical cancer (23.3%) [49]. A similar observation reported by Tang et al. revealed that the distribution of multiple HR-HPV infections declined with the increasing severity of squamous lesions, with the highest rate detected in CIN1 (33.8%), followed by CIN2 (23.6%), CIN3 (18.9%), and then cervical cancer (10.9%) [50]. A meta-analysis by Shoja et al. encompassing 5990 cases diagnosed with cervical precancer and cancer within the WHO Eastern Mediterranean Region (WEMR) found that HPV-16 was the most common type among women with precancerous (and cancerous) lesions of the cervix, and that the next most common types were HPV-6/11, 18, 52, and 56 in CIN 1/LSIL, HPV-18, 31, 6/11, and 33 in CIN 2/3, and HPV-18, 45, 31, and 35 in cancer [51]. Similar results were reported in a previously published Jordanian (local) study, which demonstrated that HPV-16 was the most prevalent type among precancerous lesions [52]. Our results are consistent with these studies, which notably showed that HPV-16 was the most prevalent type among cervical histopathological lesions.

Importantly, and slightly different from our findings, Spinillo et al. observed an increase in multiple HR-HPV infections with increasing severity of cervical neoplasia (21.6% in CIN1, 27.5% in CIN3, and 34.68% in CIN3), and found that increased risk of CIN2 and CIN3 was associated with multiple HR-HPV infections compared to a single HR-HPV infection [53]. Regarding the LR-HPV infection, in the present study, only HPV-6 and/or 11 were identified among all the precancerous lesions. This pattern of prevalence is expected, given its low carcinogenic potential, and is consistent with previous reports [54,55,56,57].

OIS is a powerful anti-tumor mechanism that mediates cell cycle arrest following aberrant activation of oncogenes [58]. OIS was first reported by Serrano et al. after the transduction of the oncogenic Ras (H-rasV12) allele into primary diploid fibroblasts [59]. Later on, OIS was shown to be a response to the activation of multiple oncogenes such as E2F, cyclin E, and Raf or, in contrast, triggered by the inhibition of tumor suppressor genes like NF1 and PTEN [60].

More relevant to this work, viral infections can trigger cellular senescence, but their specific roles in this process remain largely unknown [61,62]. As in the case of HR-HPV infection, Wells et al. reported that cellular senescence is one phenotype that is identified in HPV-positive cervical cancerous cell lines after the re-expression of exogenous HPV-18 E2 [63]. Moreover, Wells et al. observed that this E2-mediated senescent growth arrest can be overcome by the re-expression of exogenous HPV-16 E6 and E7 individually [63]. However, no previous evidence has demonstrated a connection between OIS and HR-HPV in cervical precancerous lesions in vivo.

Several in vitro studies revealed that the downregulated expression of Lamin B1 is an indicator of senescence induction [31]. The downregulation of Lamin B1, a member of the nuclear lamina, is part of the senescence-associated nuclear envelope remodeling [38]. In this study, we evaluated the expression of Lamin B1 using IHC to investigate the induction of OIS in FFPE cervical precancerous samples that were previously tested for HPV infection. We found that the expression of Lamin B1 was significantly lower in precancerous samples relative to noncancerous (chronic cervicitis) samples. Since OIS is a major phenotype identified in other precancerous lesions, our identification of Lamin B1 downregulation in cervical precancerous lesions is suggestive of its utility in identifying senescence in vivo. We confirmed this observation in other models of senescence, namely, therapy-induced senescence (TIS) in breast cancer tissue exposed to various forms of cytotoxic chemotherapy [36,40,41,42]. We have further confirmed this downregulation in Lamin B1 level at the gene expression level relative to its baseline expression in noncancerous cervical tissue.

To the best of our knowledge, Lamin B1 protein expression level has not been tested in cervical precancerous lesions. It is noteworthy that Lamin B1 expression level varies among different types of cancer tissue [64]. Moreover, a higher Lamin B1 expression level has been linked to increased cancer aggressiveness, such as in liver and pancreatic cancer [65,66], while in contrast, lower Lamin B1 is associated with poor prognosis and more aggressiveness in breast and lung cancers [67,68]. Lastly, a point of novelty of this work is that Lamin B1 downregulation is observed in precancerous cervical cells that express HPV E6 simultaneously, suggesting a possible role for HPV in eliciting a senescent response [13].

Since the identification of senescence in vivo requires the detection of several senescence-associated markers, we also investigated the expression of other senescence-related genes such as p15^INK4b^ (*CDKN2B*), p16^INK4a^ (*CDKN2A*), and p21^Cip1^ (*CDKN1A*) [31,43]. Our data suggest that both p15^INK4b^ (*CDKN2B*) and p16^INK4a^ (*CDKN2A*) are upregulated in precancerous cervical tissue when compared to noncancerous tissue. The most relevant work comes from Zhang et al. and examined the immunohistochemical expression of p15 ^INK4b^, p16^INK4a^, and p21^Cip1^ FFPE samples from 19 noncancerous, 51 precancerous, and 21 squamous cancerous tissue of the cervix [69]. Interestingly, the expression of all three cell cycle regulators was significantly higher in both CIN and cancerous samples compared to noncancerous tissue. Furthermore, the expression of p15^INK4b^ and p21^Cip1^ was significantly higher in CIN 2 compared to CIN 1, while p16^INK4a^ expression was significantly higher in CIN 3 compared to CIN 1 [69]. Unexpectedly, data from Zhang et al. did not show a significant decline in the expression of p15^INK4b^ and p16^INK4a^ in cancerous cervical tissue as an indication of senescence resolution as precancerous lesions progress into malignancy [69]. These data were confirmed by Feng et al., who showed that p15^INK4b^, p16^INK4a^, and p14^ARF^ were all overexpressed in both cervical dysplasia and carcinoma [70]. Similar results were observed in prostatic precancerous vs cancerous tissues [71].

On the other hand, Holm et al. suggested that p15^INK4b^ and p57^KIP2^ levels decline as part of vulvar epithelial transformation, indicating that markers of OIS resolve through malignant progression of gynecological cancers [72]. In this work, we have not investigated the expression of the senescence-associated β-galactosidase (SA-β-gal), which is the classical marker of senescence. This is largely due to the fact that the detection of SA-β-gal requires the availability of frozen samples [73,74,75]. Nevertheless, SA-β-gal expression is not exclusive for senescence and can be complicated by non-specificity [76,77,78]. We also have not investigated p53 expression as a senescence marker due to the higher variability in its expression in cervical tissues. For example, in almost half of the cases, p53 expression might be higher than expected in cervical cancer lesions [79,80]. This variability in p53 protein expression level certainly represents a challenge when using p53 to detect senescence in cervical lesions. Moreover, this lack of reliability of p53 as a senescence marker in cervical cancer tissue can also be observed with its downstream effector p21^Cip1^, which, in this work, showed variable expression levels among different precancerous samples, confirming its previously postulated low utility as a senescence marker in certain models [45]. In fact, despite the fact that p21^Cip1^ is readily inducible in response to DNA damage and a frequently identified marker of TIS in tumor cells in vitro [81], we have previously demonstrated its poor reliability as a senescence marker in vivo [35].

Several studies have investigated the induction of OIS in precancerous lesions, which acts as a potent barrier against tumorigenesis not only by halting the proliferation capacity of precancerous cells but also by enabling the immune clearance of these affected cells via the secretion of anti-tumorigenic SASP [82,83]. Despite the tumor-suppressive role of senescence, it has been shown that as part of malignant transformation, SASP factors can, instead, promote tumor progression [82,83]. Indeed, OIS is a double-edged sword in that it can halt tumor progression in precancerous lesions while simultaneously driving tumorigenic processes in non-transformed neighboring tissue [84]. Moreover, transcriptomic profiles of senescent cells are highly heterogeneous, generating a variable spectrum of the SASP factors that could eventually be secreted [24,85].

*MMP9* expression in our study was reduced in precancerous cervical samples in comparison to their noncancerous counterparts. Our results contradict a previous study that showed that *MMP9* is overexpressed in precancerous and cancerous cervical tissue [86]. More specifically, *MMP9* expression was higher in HSIL than in LSIL [87,88]. In another study, Sidorkiewicz et al. investigated *MMP9* expression in 31 cancerous, 17 precancerous (CIN3), and 5 ectropion cervical samples, and found that *MMP9* had higher expression in cancerous and CIN3 lesions than in the patients with ectropion [89]. One reason why we observed a relatively reduced *MMP9* expression is probably because we utilized noncancerous tissue with a chronic inflammatory state as our reference control. This might indicate that the SASP, even if robustly induced in the context of malignant transformation, is still a form of low-drive inflammatory response and not as intense as other classical forms of tissue inflammation.

In the present study, precancerous cervical samples exhibited lower levels of *IL1A* and *CCL2* expression than noncancerous, inflammatory, samples. Our findings were inconsistent with another study’s finding showing increased expression of *IL1A* in CIN compared to normal cervical tissue [90]. Mhatre et al. collected 106 cervicovaginal lavage samples from females with CIN1 and CIN3, and found that IL-1α levels were higher in CIN3 and CIN1 than in control subjects [91]. Another study found an increase in IL-1α expression in cervical cancer compared to adjacent non-tumor tissue using IHC [92]. On the other hand, Matamoros et al. showed that reduced IL-1β expression in precancerous lesions was associated with more risk of progressing into cancer [93]. It was also found that *CCL2* expression was elevated in cervical cancer [94]. In a prior study involving 93 cervical cancer tissue samples and using the RNA in situ hybridization technique, authors found that *CCL2* expression was elevated in 47 samples [95]. Furthermore, higher expression of *CCL2* in precancerous oral lesions and oral squamous cell carcinoma are often identified when compared with normal oral mucosa [96].

Another point to emphasize here is that HPV infection might have a role in influencing the expression level of these inflammatory markers (also SASP markers) independent of senescence. For example, *CXCL8* increases in the presence of E6/E7 of HPV-16 and -18 in cervical cancer samples, and *CXCL8* expression is predictive of a worse survival rate [97]. Thus, we cannot rule out the possibility that the expression of the investigated inflammatory markers in our work could be largely affected by mechanisms other than senescence. Collectively, the findings of this study highlight the heterogeneity of the SASP [24], suggesting a complex interplay between viral oncogenesis and senescence. However, this observed heterogeneity indicates the need for further investigation into the molecular drivers of this variability. Moreover, such heterogeneity may arise from differences in the HPV genotypes, the stage of infection, or the cellular microenvironment, all of which can influence the senescence response and can have various clinical outcomes [98,99]. More precisely, this variability poses challenges for the utilization of SASP-based biomarkers for senescence identification in vivo, or at least suggests that these markers cannot be examined in isolation of co-existing inflammatory states. Furthermore, this variability could cause interference when using some of the SASP components as therapeutic targets to mitigate certain deleterious effects driven by senescence [100]. Future studies should attempt to characterize the molecular mechanisms underlying this heterogeneity [101].

Senescence and autophagy are closely related processes in malignant transformation and cancer response to therapy [41,102]. Similar to senescence, in HPV-driven cervical cancer, autophagy plays a dual role, acting as both a pro-tumorigenic and tumor suppressive mechanism. Both HPV oncoproteins, E6 and E7, can result in the suppression of key autophagic processes, leading to the promotion of cellular proliferation and immunoevasion [103]. Autophagy also facilitates metabolic adaptation in rapidly proliferating HPV-infected, which propagates tumor growth and survival [104]. However, in certain models, defective autophagy may enhance the progression of HPV-infected lesions [104]. These mechanisms highlight the complex interplay between autophagy and HPV infection and suggest a link between HPV, autophagy, and senescence in the pathogenesis of cervical cancer [105].

Lastly, we acknowledge that the small sample size of our study is a major limitation. This prevented us from conducting a comparison of the expression of OIS markers between CIN 1, CIN 2, and CIN 3, as reported previously in oral or epidermal epithelial dysplasia [106]. This is, indeed, attributed to the lower prevalence of HPV infection in Jordan, where this study was conducted [26]. In all cases, our data should only be viewed in the context of this particular sample, and further investigation of the role of senescence in propagating cervical cancer is necessary. These results also confirm the variability of detecting senescence markers in vivo and the requirement for better detection approaches [107].

## Figures and Tables

**Table 1:** Primers’ sequences utilized for qrtPCR. Sequences are in 5′ → 3′.

Gene	Forward	Reverse	Ref
*CDKN1A*	GAGGCCGGGATGAGTTGGGAGGAG	CAGCCGGCGTTTGGAGTGGTAGAA	[29]
*CDKN2A*	GGGTCGGGTAGAGGAGGTG	CATCATGACCTGGATCGGC	[30]
*CDKN2B*	GACCGGGAATAACCTTCCAT	CACCAGGTCCAGTCAAGGAT	[30]
*LMNB1*	GTATGAAGAGGAGATTAACGAGAC	TACTCAATTTGACGCCCAG	[31]
*MMP9*	CAGTCCACCCTTGTGCTCTTC	TGCCACCCGAGTGTAACCAT	[32]
*IL1A*	AGAGGAAGAAATCATCAAGC	TTATACTTTGATTGAGGGCG	[31]
*CCL2*	GATCTCAGTGCAGAGGCTCG	TGCTTGTCCAGGTGGTCCAT	[31]
*CXCL8*	AAGAGCCAGGAAGAAACCACC	CTGCAGAAATCAGGAAGGCTG	[33]
*TGFB1*	TCGCCAGAGTGGTTATCTT	TAGTGAACCCGTTGATGTCC	[34]
*GAPDH*	AGCCACATCGCTCAGACAC	GCCCAATACGACCAAATCC	[31]

Abbreviations: Ref: reference.

## Data Availability

All data analyzed during this study are included in this published article, while all raw data generated as part of this work are available from the corresponding author on reasonable request.
